# Development and evaluation of a triplex droplet digital PCR method for differentiation of *M. tuberculosis*, *M. bovis* and BCG

**DOI:** 10.3389/fmicb.2024.1397792

**Published:** 2024-06-14

**Authors:** Yao Qu, Mengda Liu, Xiangxiang Sun, Yongxia Liu, Jianzhu Liu, Liping Hu, Zhiqiang Jiang, Fei Qi, Wenlong Nan, Xin Yan, Mingjun Sun, Weixing Shao, Jiaqi Li, Shufang Sun, Haobo Zhang, Xiaoxu Fan

**Affiliations:** ^1^National Animal Tuberculosis Reference Laboratory, Division of Zoonoses Surveillance, China Animal Health and Epidemiology Center, Qingdao, Shandong, China; ^2^College of Animal Technology, Shandong Agricultural University, Taian, Shandong, China; ^3^Key Laboratory of Major Ruminant Infectious Disease Prevention and Control (East) of Ministry, Agriculture and Rural Affairs, Qingdao, Shandong, China; ^4^Key Laboratory of Animal Biosafety Risk Warning Prevention and Control (South) of Ministry, Agriculture and Rural Affairs, Qingdao, Shandong, China; ^5^Shandong Center for Animal Disease Prevention and Control, Jinan, Shandong, China

**Keywords:** molecular diagnosis, multiplex droplet digital PCR, tuberculosis, *M. tuberculosis*, *M. bovis*, BCG

## Abstract

**Introduction:**

Tuberculosis, caused by *Mycobacterium tuberculosis* complex (MTBC), remains a global health concern in both human and animals. However, the absence of rapid, accurate, and highly sensitive detection methods to differentiate the major pathogens of MTBC, including *M. tuberculosis*, *M. bovis*, and BCG, poses a potential challenge.

**Methods:**

In this study, we have established a triplex droplet digital polymerase chain reaction (ddPCR) method employing three types of probe fluorophores, with targets *M. tuberculosis* (targeting CFP-10-ESAT-6 gene of RD1 and Rv0222 genes of RD4), *M. bovis* (targeting CFP-10-ESATs-6 gene of RD1), and BCG (targeting Rv3871 and Rv3879c genes of ΔRD1), respectively.

**Results:**

Based on optimization of annealing temperature, sensitivity and repeatability, this method demonstrates a lower limit of detection (LOD) as 3.08 copies/reaction for *M. tuberculosis*, 4.47 copies/reaction for *M. bovis* and 3.59 copies/reaction for BCG, without cross-reaction to *Mannheimia haemolytica*, *Mycoplasma bovis*, *Haemophilus parasuis*, *Escherichia coli*, *Pasteurella multocida*, *Ochrobactrum anthropi*, *Salmonella choleraesuis*, *Brucella melitensis*, and *Staphylococcus aureus*, and showed repeatability with coefficients of variation (CV) lower than 10%. The method exhibits strong milk sample tolerance, the LOD of detecting in spike milk was 5 × 10^3^ CFU/mL, which sensitivity is ten times higher than the triplex qPCR. 60 clinical DNA samples, including 20 milk, 20 tissue and 20 swab samples, were kept in China Animal Health and Epidemiology Center were tested by the triplex ddPCR and triplex qPCR. The triplex ddPCR presented a higher sensitivity (11.67%, 7/60) than that of the triplex qPCR method (8.33%, 5/60). The positive rates of *M. tuberculosis*, *M. bovis*, and BCG were 1.67, 10, and 0% by triplex ddPCR, and 1.67, 6.67, and 0% by triplex qPCR, with coincidence rates of 100, 96.7, and 100%, respectively.

**Discussion:**

Our data demonstrate that the established triplex ddPCR method is a sensitive, specific and rapid method for differentiation and identification of *M. tuberculosis*, *M. bovis*, and BCG.

## Introduction

Tuberculosis (TB) is a zoonotic disease that leads to the formation of caseous necrotic nodules in multiple organs of both humans and animals (Vielmo et al., 2020). According to the WHO TB report 2022, there were 10.6 million new cases of tuberculosis and 1.6 million tuberculosis-related deaths in 2021 (World Health Organization, 2022). TB is primarily caused by the *Mycobacterium tuberculosis* complex (MTBC), which consists of several members including the *Mycobacterium tuberculosis* (*M. tuberculosis*), *Mycobacterium bovis* (*M. bovis*) and Bacillus Calmette-Guérin (BCG) (Lekko et al., 2020). Despite the genetic similarity, ranging from 99.97 to 99.99%, these members are different microorganisms exhibit different host preferences and pathogenicity resulting in a limited availability of methods for making a differential diagnosis (Pinsky and Banaei, 2008; Kanabalan et al., 2021). MTBC can infect humans and a variety of animals, posing a threat to the concept of “one health” (Marais et al., 2019). For MTBC not only effects domestic animals such as cattle (Gutierrez Reyes et al., 2012) and goats (Quintas et al., 2010), companion animals such as cats (Cerna et al., 2019) and dogs (Rocha et al., 2017), but also affects wildlife including elephants (Maslow and Mikota, 2015), badgers (Smith and Budgey, 2021), deer (Amato et al., 2016), etc. Notably, *M. tuberculosis* is the primary pathogen responsible for human TB, resulting in millions of deaths annually (Rahlwes et al., 2023). While human TB is primarily caused by *M. tuberculosis* (Ehrt et al., 2018), a small percentage is attributed to *M. bovis* due to their high genetic similarity, with approximately 0.5–7% of cases resulting from human contact with infected cattle or related products (Vayr et al., 2018). Furthermore, BCG remains the sole TB vaccine available since the 20th century (Tran et al., 2014). Already, 4 billion people have been vaccinated against TB with the BCG vaccine, resulting in a 60–80% reduction in the incidence of active TB (Kuan et al., 2020). Although BCG greatly reduces the virulence of *M. bovis* as a live vaccine, it occasionally causes local or disseminated disease in immunocompromised individuals (World Health Organization, 2020). Extensive research has been conducted on BCG immunization in domestic and wild animals (such as badgers) over the past 10–20 years. While it may not complete prevent the occurrence of TB, the protection it provides could significantly reduce transmission from infected animals to other animals (Buddle et al., 2018).

In recent years, *M. bovis* has shown a tendency of extensive and multi-drug resistance. The treatment protocols of tuberculosis caused by *M. bovis* and *M. tuberculosis* should be differentiated (El-Sayed et al., 2016). Compared with *M. tuberculosis* and *M. bovis*, there are 2,437 SNPs differences (Garnier et al., 2003). The emergence of point mutations in *M. bovis* could result in the development of drug resistance, with drug-resistant mutants potentially proliferating due to irregular medication in cattle feeding, ultimately leading to multiple drug resistance. This scenario poses significant challenges to the effective treatment of TB resulting from *M. bovis* infection in humans, particularly considering the increased difficulty in treating *M. bovis* strains compared to *M. tuberculosis* due to their multiple drug resistance. As a result, early identification of the pathogen during infection becomes paramount (Kabir et al., 2020; Vazquez-Chacon et al., 2021; Dos Anjos et al., 2022). Moreover, by accurately distinguishing between infections caused by *M. tuberculosis*, *M. bovis*, and the BCG strains, the clinical epidemiology of bovine tuberculosis can be improved (Pfeiffer, 2013). All in all, rapid and accurate identification of these bacteria from suspected samples is crucial for early pathogen identification, contact tracing, detecting latent infection, distinguishing nature infection with vaccine immunity, and differential diagnosis. Despite their differences of genome less than 0.05%, these strains can be distinguished based on their different characteristics (Bigi et al., 2016). Comparative genomic analysis of *M. tuberculosis*, *M. bovis*, and BCG strains has revealed the presence or absence of certain regions of difference (RDs) in their genomes (Brosch et al., 2007; Bespiatykh et al., 2021). Notably, RD1 is absent in all BCG strains, resulting in a deletion of approximately 9.5kb, forming ΔRD1. Additionally, the RD4 fragment was absent in all *M. bovis* strains and BCG strains. The presence of these RDs distinguishes *M. tuberculosis* (RD1 and RD4), from *M. bovis* (RD1), and BCG strains (ΔRD1).

Droplet Digital Polymerase Chain Reaction (ddPCR), a third-generation PCR technique, has emerged as an advancement of the traditional PCR method, enabling the absolute quantification of nucleic acids through the isolation and amplification of individual DNA molecules and calculated by Poisson distribution (Li et al., 2018). In comparison to PCR and quantitative polymerase chain reaction (qPCR), ddPCR demonstrates unique sensitivity for samples with low copy numbers and overcomes the limitations of standard curves, leading to higher accuracy (Huggett and Whale, 2013). Additionally, multiplex ddPCR allows for the simultaneous detection of multiple targets using multiple fluorescent channels, while maintaining high sensitivity and specificity (Ganova et al., 2021). Previous studies have showcased the superior detection capabilities of multiplex ddPCR in complex matrices such as food (McMahon et al., 2017), fecal matter (Chen et al., 2023), aquaculture water (Lewin et al., 2020), and mutation detection with extremely low DNA concentrations (de Kock et al., 2021). Therefore, multiplex ddPCR has been identified as a crucial direction for future diagnostic methods. While the utilization of ddPCR in diagnosing *M. tuberculosis* in infected humans (Lyu et al., 2020) and macaques (Song et al., 2018) have been demonstrated, and PCR has been employed in the differential diagnosis of MTBC pathogens (Krysztopa-Grzybowska et al., 2014), few studies on dPCR have specifically addressed the differential diagnosis of pathogens within MTBC. *M. tuberculosis, M. bovis*, and other numbers of MTBC such as *M. canetti* share a common progenitor. Through evolutionary processes, their genomes have occurred mutations that facilitate for inter-species transmission, leading to the formation of regions of difference known as RDs. Compared to *M. tuberculosis*, *M. africanum* exhibits the absence of region RD9 and the presence of region TbD1. *M. microti* demonstrates deletions in RD7, RD9 and RD10, along with specific absences in called RD1^mic^, RD5^mic^, MiD1, MiD2 and MiD3, as compared to *M. tuberculosis*. *M. caprae* is characterized by the absence of RD7-RD10, RD12 and RD13, with 1,577 gene variants distinguishing it from *M. tuberculosis*. *M. bovis* exhibits the absence of RD4-RD10 compared to *M. tuberculosis*, and BCG further lacks RD1-RD3 based on *M. bovis.* Moreover, *M. bovis* and BCG possess TbD1, which is not present in *M. tuberculosis*. *M. pinnipedii* lacks RD7-RD10 and is missing PiD1 and PiD2 compared to *M. tuberculosis.* Lastly, *M canettii* has all RD regions except phiRv1, phiRv2, and a segment of RD12 (Gonzalo-Asensio et al., 2014; Malone and Gordon, 2017; Kanipe and Palmer, 2020; Romano et al., 2022). Consequently, various methods can be established to distinguish members of MTBC based on these distinctive RDs.

In this study, we have developed a triplex ddPCR-based method for highly sensitive and simultaneous differential detection of *M. tuberculosis*, *M. bovis*, and BCG. The three target genes or fragments are concurrently detected using three fluorescent probes: 6-Carboxyfluorescein (FAM), 5-VIC phosphoramidite (VIC), and Cy5 phosphoramidite (CY5), within a five-color ddPCR system (Sniper DQ24pro™). This method was comprehensively evaluated alongside qPCR methods. Its ability to identify three pathogens DNA in milk samples was tested, demonstrating its suitability for rapid and sensitive detection of bio-threatening pathogens in suspicious milk samples. Although the high level of genetic similarity within the MTBC complex poses a challenge in distinguishing between different species, the advancement of ddPCR methods that target specific genetic regions, the accurate diagnosis of *M. tuberculosis*, *M. bovis*, and BCG has become achievable, resulting in an anticipated improvement in the differential diagnosis of TB in the future.

## Materials and methods

### Genomic DNA samples and inactivated bacteria samples

All DNA samples, including *M. tuberculosis* C2, *M. bovis* XJ/18/97 (Xu et al., 2021) and BCG Tokyo 172, *Mannheimia haemolytica*, *Mycoplasma bovis*, *Haemophilus parasuis*, *Escherichia coli*, *Pasteurella multocida*, *Ochrobactrum anthropi*, *Salmonella choleraesuis*, *Brucella melitensis* and *Staphylococcus aureus* used in this study were obtained among previous studies and kept in the National Animal Tuberculosis Reference Laboratory of China Animal Health and Epidemiology Center (Qingdao, China). The DNA and plasmids used in this study are listed in Supplementary Table 1. The DNA extraction method is detailed in the Supplementary Material.

Inactivated bacteria (*M. tuberculosis* C2, *M. bovis* XJ/18/97 and BCG Tokyo 172) in spiked milk and water were also kept in the National Animal Tuberculosis Reference Laboratory of China Animal Health and Epidemiology Center (Qingdao, China). The concentration ranged from 5 × 10^1^ to 5 × 10^6^ CFU/mL. DNA from spiked milk and water samples were extracted using a Milk Bacterial DNA Isolation kit (Norgen Biotek, Canada).

### Primers and probes

According to previous studies, there are 16 different regions of MTBC (RD1-16) (Qu et al., 2020), RD1 is present in *M. tuberculosis* and *M. bovis*, while RD4 solely exists in *M. tuberculosis*. Moreover, the presence of ΔRD1 is exclusive to BCG strains. Thus, triplex ddPCR relies on the targeting of specific sequences, namely CFP-10 and ESAT-6 of RD1, Rv0222 of RD4, as well as the upstream Rv3871 and downstream Rv3879c of ΔRD1. The design of all primers and Taqman^®^ probes was carried out using the PrimerQuest™ Tool (Integrated DNA Technologies, US), while their synthesis was performed by Shanghai Sangon Biotech (China). The RD1 probe was labeled with FAM, the RD4 probe with VIC, and the ΔRD1 probe with CY5. All primers and Taqman^@^ probes used in this study is shown in Supplementary Table 2.

### Preparation of recombinant standard plasmids

Recombinant standard plasmids were engineered to contain specific regions: 623bp of RD1, 789bp of RD4, and 1000bp (500bp upstream Rv3871 and 500bp downstream Rv3879c) of ΔRD1. These plasmids were individually constructed within the pUC57 vector and designated as p-RD1, p-RD4, and p-ΔRD1. All recombinant standard plasmids were synthesized by Shanghai Sangon Biotech (China). The concentrations of these plasmids were determined to be 4.8 × 10^9^ copies/μL and were stored at −20°C until required for use. Plasmids were isolated from *E. coli* DH5α culture medium using an E.Z.N.A. HP Plasmid DNA Mini kit (Omega Bio-Tek, US).

### Real-time quantitative PCR

The qPCR mixture consisted of 10 μL of Takara Premix Ex Taq™ Probe qPCR Mix, along with 400 nM primers, 200 nM probes, 1 μL DNA sample, and nuclease-free water to reach a final volume of 20 μL. The thermal cycling program was set at 95°C for 10 min, followed by 40 cycles of denaturation at 94°C for 15 s and annealing at 60°C for 30 s. All qPCR reactions were conducted using the Applied Biosystems QuantStudio 5 system (Thermo Fisher United States).

### Triplex droplet digital PCR

The triplex ddPCR reaction mixtures were prepared by combining 11 μL of 2 × dPCR probe Master mix plus (Sniper, China), along with 455 nM specific primers for each target, 150 nM RD1 probe (FAM-labeled), 500 nM RD4 probe (VIC-labeled), 250 nM ΔRD1 probe (CY5-labeled), 1 μL DNA sample, and nuclease-free water to a final volume of 22 μL. All ddPCR assays were performed using the Sniper DQ24pro™ dPCR systems, which includes a droplet generator and an automated reader. The reaction mixture was transferred to the internal stent of the integrated machine, followed by the installation of the droplet reaction plate, droplet reaction plate cover, and droplet generation kit onto the corresponding stent. The droplet generation oil was connected to the integrated machine, generating up to 20,000 nanoliter-sized droplets. The thermal cycle program was performed for 5 min at 60°C with a ramp rate of 2°C/s at each step; followed by 40 cycles of 95°C for 20 s, 60°C for 30 s, and held at 12°C. Data analysis was performed using the SightPro (x64) software.

### Analysis of sensitivity, specificity and repeatability of the triplex ddPCR

The sensitivity of the assay was assessed using bacteria DNA and standard plasmids. Bacteria DNA were diluted in nuclease-free water, ranging from 3 × 10^4^ to 3 × 10^0^ copies/μL, while standard plasmids were diluted in nuclease-free water, ranging from 4.8 × 10^5^ to 4.8 × 10^0^ copies/μL. The bacteria DNA samples were tested in triplicate using the triplex ddPCR assay, while the standard plasmid samples were tested using the single-target ddPCR assay. To determine the limit of blank (LOB) for each channel, 8 nuclease-free water samples were tested as blank samples based on a previous study. Samples with copy numbers above the LOB were considered positive, which was calculated as LOB = mean_blank_ + 1.645 (SD_blank_). The lowest DNA concentration that could be detected was defined as the limit of detection (LOD). Quantitative curves were constructed for each target, with log_10_ (theoretical copies/reaction) as the *x*-axis, and log_10_ (copies/reaction measured) or Ct value as the *y*-axis. The linear fitting coefficient (R^2^) was calculated using GraphPad Prism 10.0.

The specificity of the assay was assessed using a total of 9 other pathogenic bacteria, including *Mannheimia haemolytica*, *Mycoplasma bovis*, *Haemophilus parasuis*, *Escherichia coli*, *Pasteurella multocida*, *Ochrobactrum anthropi*, *Salmonella choleraesuis*, *Brucella melitensis*, and *Staphylococcus aureus*. Each DNA was tested three times independently.

The bacterial DNA samples with different concentrations of 3 × 10^4^, 3 × 10^3^, 3 × 10^2^ copies/μL were tested 8 times to determine the coefficient of variation (CV) for intra-assay repeatability.

### Detection of clinical samples

All clinical DNA samples, including 20 milk, 20 tissue and 20 from swab samples were extracted in previous studies and kept in the National Animal Tuberculosis Reference Laboratory of China Animal Health and Epidemiology Center (Qingdao, China), and tested by the established triplex ddPCR and the triplex qPCR methods. In each reaction, nuclease-free water was used as a negative control, while *M. tuberculosis*, *M. bovis*, and BCG DNA served as positive controls. The DNA extraction method is detailed in the Supplementary Material.

## Results

### Optimization of reaction conditions for establishing the triplex ddPCR

**FIGURE 1:**
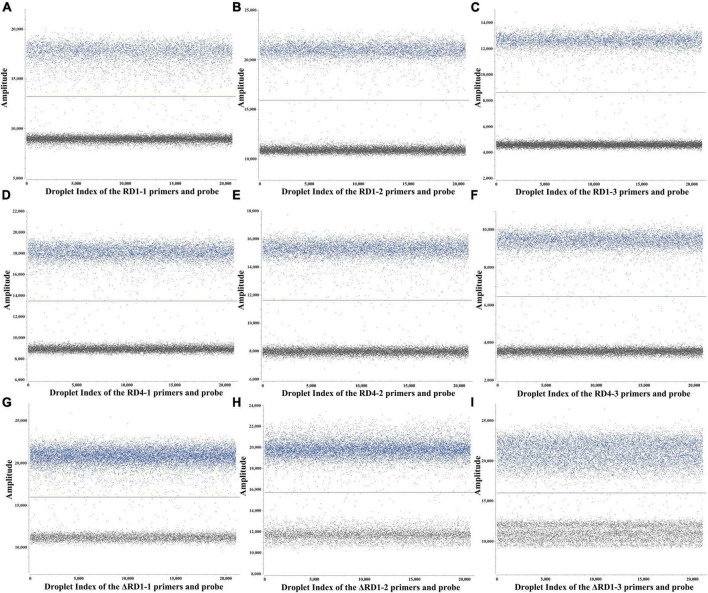
ddPCR assay for primers and probes screening. **(A–C)** 3 sets primers and probes screening for RD1. **(D–F)** 3 sets primers and probes screening for RD4. **(G–I)** 3 sets primers and probes screening for ΔRD1.

The p-RD1, p-RD4, and p-ΔRD1 standard plasmids were utilized to optimize the primers and probe sets, annealing temperature, probe concentrations of the triplex ddPCR. To accomplish this, the standard plasmids were 10-fold serially diluted, ranging from 4.8 × 10^7^ to 4.8 × 10^0^ copies/μL for each plasmid, and served as the template. For each target, three sets of primers and probes were designed (Supplementary Table 2), and the best set was determined using both qPCR and ddPCR methods. The qPCR results revealed that primers and probes from RD1 set3, RD4 set3 and ΔRD1 set2 exhibited superior amplification curves and the highest fluorescence amplitude (Supplementary Figure 1). For ddPCR, each plasmid with a concentration of 4.8 × 10^2^ copies/μL, the result showed that the most noticeable difference in fluorescence amplitude between negative and positive droplets was observed within the same sets of primers and probes, and the number of positive droplets was the highest (Figure 1). Consequently, RD1 set3, RD4 set3, and ΔRD1 set2 were selected as the primer and probe combinations for further experiments (Table 1).

**TABLE 1:** Primers and probes sequences were chosen for three targets.

Target gene	RD region	Fragment length	Design sequence 5′–3′
CFP-10 to ESAT-6	RD1	623	F:CCTCGCAAATGGGCTTCT
			R:GACGTGACATTTCCCTGGATT
			P:FAM-AGTGGAATTTCGCGGGTATCGAGG-DHQ1
Rv0222	RD4	789	F:TATGCGATAGCCATGGAGTTG
			R:CCATTGGCGGTGATCTTCT
			P:VIC-TCGATGCTGCGATCGCGTTG-DHQ1
Rv3871 and Rv3879c	ΔRD1	996	F:GGATTTGACGTCGTGCTTCT
			R:CGATCTGGCGGTTTGGG
			P:CY5-ATCCAGCATCTGTCTGGCATAGCT-DHQ2

**FIGURE 2:**
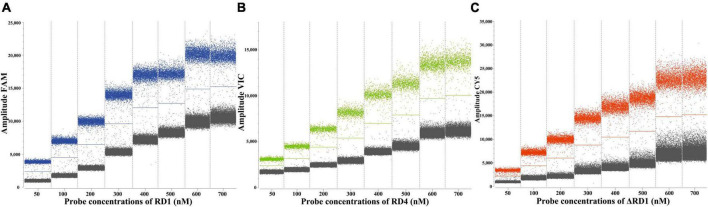
Determination of the optimal probe concentrations of the ddPCR of RD1 **(A)**, RD4 **(B)**, ΔRD1 **(C)**.

The primer and probe concentrations were optimized using standard plasmids, each with a concentration of 4.8 × 10^3^ copies/μL. The arrangement and combination of different concentrations of primers and probes were analyzed using SightPro software (Sniper Technologies, China). The concentration combinations that displayed the most pronounced fluorescence amplitude interval between negative (gray) and positive (color) with distinct boundaries were determined as the optimal primer and probe concentrations (Figure 2). The optimal probe concentrations, detailed in Table 2, were determined to be 150 nM for RD1, 500 nM for RD4, and 250 nM for ΔRD1.

**TABLE 2:** The reaction mix of the triplex ddPCR and the triplex qPCR.

	Triplex ddPCR reaction		Triplex qPCR reaction	
	Volume (μL)	Final concentration (nM)	Volume (μL)	Final concentration (nM)
2 × dPCR probe Master mix	11	1×	/	/
2 × Premix Ex Tag (Probe qPCR)	/	/	10	1×
pRD1-F(10 μM)	1	455	0.8	400
pRD1-R(10 μM)	1	455	0.8	400
pRD1-P(10 μM)	0.33	150	0.4	200
pRD4-F(10 μM)	1	455	0.8	400
pRD4-R(10 μM)	1	455	0.8	400
pRD4-P(10 μM)	1.1	500	0.4	200
pΔRD1-F(10 μM)	1	455	0.8	400
pΔRD1-R(10 μM)	1	455	0.8	400
pΔRD1-P(10 μM)	0.55	250	0.4	200
Template	1	/	1	/
RNase Free H_2_O	Up to 22	/	Up to 20	/

**FIGURE 3:**
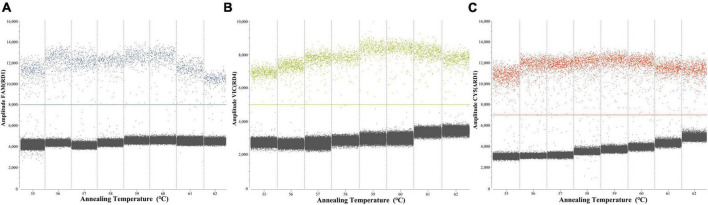
Screening the optimum annealing temperature from 55 to 62°C of RD1 **(A)**, RD4 **(B)**, ΔRD1 **(C)**.

To determine the optimal ddPCR annealing temperature, each plasmid with a concentration of 4.8 × 10^2^ copies/μL was utilized at annealing temperatures from 55 to 62°C. The result showed that the optimal annealing temperature was 60°C, which could generate the highest fluorescence amplitude (Figure 3).

After optimizing the reaction conditions, the triplex ddPCR assay was successfully established (Table 2). The total volume of the 22 μL reaction mixtures consisted of 11 μL of Sniper 2 × dPCR probe Master mix plus (Sniper Biotechnology, China), 1 μL of each primer RD1 F/R (10 μM), 0.33 μL of probe RD1-P (10 μM), 1 μL of each primer RD4 F/R (10 μM), 1.1 μL of probe RD4-P (10 μM), 1 μL of each primer ΔRD1 F/R (10 μM), 0.55 μL of probe ΔRD1-P (10 μM), 1 μL of DNA template, and 2.02 μL of nuclease-free water. The ddPCR amplifications were conducted as follows: initial denaturation at 60°C for 5 min, denaturation at 95°C for 5 min, followed by 40 cycles of 95°C for 20 s and 60°C for 30 s. Subsequent to amplification, the absolute copies of each sample were automatically reported by the Sniper System.

### Evaluation of the triplex ddPCR method with DNA samples

**FIGURE 4:**
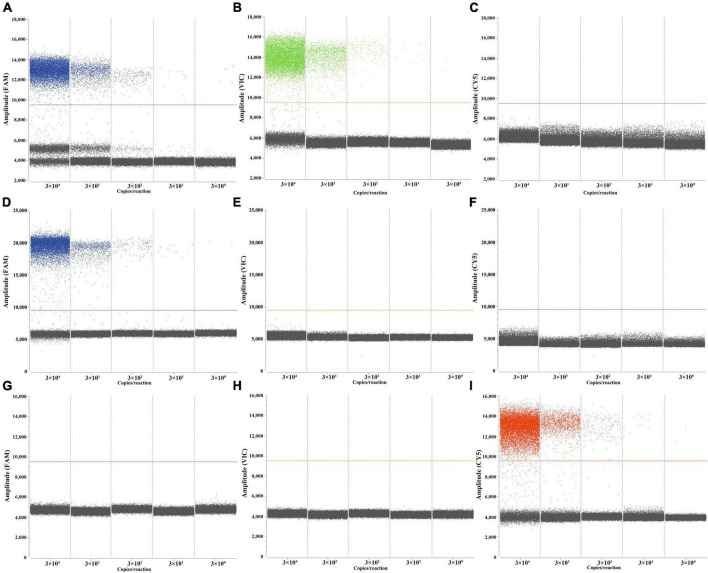
Performances of the triplex ddPCR assay by target DNA from 3 × 10^4^ to 3 × 10^0^ copies/μL. **(A–C)** Detection results of the target *M. tuberculosis*. **(D–F)** Detection results of the target *M. bovis*. **(G–I)** Detection results of the target BCG.

**FIGURE 5:**
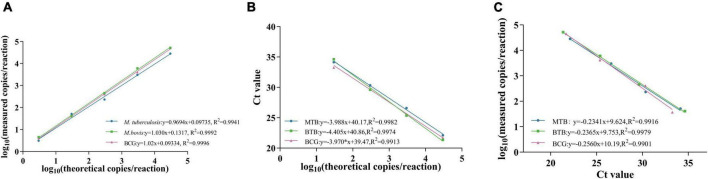
Standard curve of *M. tuberculosis*, *M.bovis* and BCG. **(A,B)** show the standard curves of triplex ddPCR and triplex qPCR, respectively, and **(C)** indicates the correlation between them.

LOBs were established by testing eight blank samples and calculating the average and standard deviation (SD) of their copy numbers, with a confidence interval set at 95%. The determined LOBs for the FAM, VIC, and CY5 channels in the blank samples were 1.07, 0.74, and 1.31 copies/reaction, respectively. Subsequently, copies below the LOBs in the experiments were considered negative. The sensitivity of the triplex ddPCR assay for each target was assessed by testing a range of diluted *M. tuberculosis*, *M. bovis*, and BCG DNA solutions (from 3 × 10^4^ to 3 × 10^0^ copies/μL). When the test yields positive results for both RD1 and RD4, it indicates the sample is *M. tuberculosis*. If only RD1 is positive, the sample is *M. bovis*, and only when ΔRD1 is positive, it signifies BCG. The results indicated that the LODs for *M. tuberculosis* were 3.08 copies/reaction, for *M. bovis* were 4.47 copies/reaction, and for BCG were 3.59 copies/reaction (Figure 4). These results indicate that accurate detection can be achieved when the target gene content in the sample is above the LOD. Quantitative curves were generated with log_10_ (theoretical copies/reaction) plotted on the *x*-axis and log_10_ (copies/reaction measured) plotted on the *y*-axis. Each target exhibited a strong quantitative linearity (R^2^> 0.99) within the theoretical range of 3 × 10^0^ to 3 × 10^4^ copies/reaction (Figure 5).

Specificity tests were performed using 9 nucleic acids from other pathogens, including *Mannheimia haemolytica*, *Mycoplasma bovis*, *Haemophilus parasuis*, *Escherichia coli*, *Pasteurella multocida*, *Ochrobactrum anthropi*, *Salmonella choleraesuis*, *Brucella melitensis*, and *Staphylococcus aureus*. As depicted in Supplementary Figure 2, no cross-amplification was observed for these bacterial DNA (3 × 10^2^ copies/μL), confirming the specificity of our triplex ddPCR method (Supplementary Figure 2).

Three concentrations of 3 × 10^3^ to 3 × 10^1^ copies/μL for each bacteria DNA were used as templates to evaluate the repeatability. The results showed that the CVs of intra-assay ranged from 1.93 to 4.74%, 0.92–9.15%, and 3.13–9.26%, respectively (Table 3).

**TABLE 3:** Analysis of the repeatability of the triplex ddPCR.

DNA	Final concentration (copies/μL)	Repeatability assay		
		SD	AVG	CV%
*M. tuberculosis*	3 × 10^4^	283.18	14646.28	1.93%
	3 × 10^3^	74.68	1574.18	4.74%
	3 × 10^2^	6.17	175.95	3.50%
*M. bovis*	3 × 10^4^	178.50	19320.18	0.90%
	3 × 10^3^	29.31	1338.84	2.19%
	3 × 10^2^	10.76	117.62	9.15%
BCG	3 × 10^4^	769.98	24532.97	3.14%
	3 × 10^3^	98.09	2365.36	4.15%
	3 × 10^2^	22.10	238.48	9.27%

### Comparison analysis of the sensitivity and standard curves between the triplex ddPCR and triplex qPCR

Sensitivity tests were performed on *M. tuberculosis*, *M. bovis*, and BCG DNA templates from 3 × 10^4^ to 3 × 10^0^ copies/μL using triplex ddPCR and triplex qPCR. The results revealed that ddPCR could detect samples containing as few as 3 × 10^0^ copies of the target DNA, whereas qPCR could only detect samples containing 3 × 10^1^ copies of the target DNA (Table 4). The correlation coefficients between the triplex ddPCR and the triplex qPCR were 0.9916 for *M. tuberculosis*, 0.9979 for *M. bovis*, and 0.9901 for BCG (Figure 5), indicating a positive association between these two methods.

**TABLE 4:** Comparison analysis of the sensitivity between the triplex ddPCR and triplex qPCR assay.

Theoretical copies/μl	Copies/reaction in triplex ddPCR			CT in triplex qPCR		
	*M. tuberculosis*	*M. bovis*	BCG	*M. tuberculosis*	*M. bovis*	BCG
3 × 10^4^	2.8 × 10^4^	5.2 × 10^4^	4.5 × 10^4^	22.10	21.35	21.67
3 × 10^3^	3.0 × 10^3^	6.0 × 10^3^	4.1 × 10^3^	26.59	25.39	25.36
3 × 10^2^	2.3 × 10^2^	4.5 × 10^2^	4.1 × 10^2^	30.34	29.62	30.32
3 × 10^1^	5.1 × 10^1^	4.1 × 10^1^	3.7 × 10^1^	34.15	34.62	33.25
3 × 10^0^	3.1 × 10^0^	4.5 × 10^0^	3.6 × 10^0^	NA	NA	NA

The performance of comparison analysis was also evaluated using known bacterial concentrations in spiked milk and water samples to mimic real clinical samples. The spiked milk samples and water at concentrations of 5 × 10^3^ and 5 × 10^2^ CFU/mL could be identified by triplex ddPCR, respectively. In contrast, triplex qPCR could detect concentration of 5 × 10^4^ and 5 × 10^3^ CFU/mL for spiked milk and water samples, respectively (Tables 5, 6). These results all suggested that the sensitivity of triplex ddPCR was ten times higher than the triplex qPCR.

**TABLE 5:** Estimated the sensitivity of three bacteria in spiked nuclease-free water samples by triplex ddPCR.

CFU/mL	Copies/reaction in triplex ddPCR			CT in triplex qPCR		
	*M. tuberculosis*	*M. bovis*	BCG	*M. tuberculosis*	*M. bovis*	BCG
5 × 10^6^	5.3 × 10^4^	1.0 × 10^5^	2.5 × 10^4^	19.91	21.15	21.00
5 × 10^5^	6.3 × 10^3^	9.7 × 10^3^	2.5 × 10^3^	23.94	25.13	25.33
5 × 10^4^	5.6 × 10^2^	6.9 × 10^2^	1.7 × 10^2^	28.17	29.56	29.04
5 × 10^3^	4.1 × 10^1^	6.7 × 10^1^	1.0 × 10^1^	32.34	34.43	33.25
5 × 10^2^	1.8 × 10^0^	6.7 × 10^0^	3.1 × 10^0^	NA	NA	NA
5 × 10^1^	NA	NA	NA	NA	NA	NA

**TABLE 6:** Estimated the sensitivity of three bacteria in spiked milk samples by triplex ddPCR.

CFU/mL	Copies/reaction in triplex ddPCR			CT in triplex qPCR		
	*M. tuberculosis*	*M. bovis*	BCG	*M. tuberculosis*	*M. bovis*	BCG
5 × 10^6^	6.1 × 10^3^	2.2 × 10^3^	1.9 × 10^3^	24.24	26.72	25.56
5 × 10^5^	3.6 × 10^2^	1.7 × 10^2^	1.5 × 10^2^	27.26	29.65	29.77
5 × 10^4^	3.3 × 10^1^	1.7 × 10^1^	2.1 × 10^1^	32.09	33.41	34.17
5 × 10^3^	5.9 × 10^0^	3.0 × 10^0^	4.1 × 10^0^	NA	NA	NA
5 × 10^2^	NA	NA	NA	NA	NA	NA
5 × 10^1^	NA	NA	NA	NA	NA	NA

### Clinical performance of triplex ddPCR

The 60 clinical DNA samples were tested using the triplex ddPCR and the triplex qPCR. The positive rates of *M. tuberculosis*, *M. bovis*, and BCG were 1.67%, 10% and 0%, respectively. In comparison, 1.67%, 6.67% and 0% from the triplex qPCR results, respectively. The results suggested that the sensitivity of the triplex ddPCR were higher than the triplex qPCR, with the coincidence rates of *M. tuberculosis*, *M. bovis*, and BCG were 100%, 96.7% and 100%, respectively (Table 7).

**TABLE 7:** Clinical results for triplex ddPCR.

	Detection results (positive samples/total samples)		
Detection method	*M. tuberculosis*	*M. bovis*	BCG
ddPCR	1/60	6/60	0/60
qPCR	1/60	4/60	0/60
Coincidence rates	100%	96.7%	100%

## Discussion

Prior to the COVID-19 outbreak, tuberculosis (TB) had the highest mortality rate among single infectious diseases (World Health Organization, 2021). Timely identification of infecting strains and early-stage diagnosis of TB could control the source of infection and enable the implementation of targeted prevention and treatment measures. However, due to the high genetic similarity within MTBC, differentiating its members presents a challenge. After years of research, the discovery of RDs has helped us understand the genetic differences within MTBC genomes, allowing us to distinguish among *M. tuberculosis*, *M. bovis* and BCG strains. In this study, we developed and evaluated a triplex ddPCR method for the identification of *M. tuberculosis*, *M. bovis* and BCG using RD1, RD4, and ΔRD1 with a three-color ddPCR system. For the first time, the triplex ddPCR method for differentially detecting *M. tuberculosis*, *M. bovis* and BCG was successfully developed, and has the potential to be used in the differential identification of MTBC in early or latent TB infection due to its high sensitivity and accuracy.

Conventional differentiation of members within MTBC relies on a combination of tests that assess the growth characteristics and biochemical properties of the strains. However, this approach is time-consuming, taking 2–3 weeks, and can sometimes yield indeterminate results (Bolanos et al., 2017). Additionally, molecular methods like PCR have been developed for differentiating members of MTBC. Although PCR allows for qualitative analysis, its sensitivity limits prevent accurate quantification and early diagnosis of TB (Owusu et al., 2023). In the initial or latent stages of infection, the qPCR method is constrained by standard curve and Ct value considerations, as well as its susceptibility to PCR reaction inhibitors, thus limiting its ability to detect very low sample concentrations. Conversely, ddPCR makes use of the Poisson distribution to determine positive sample copy numbers, allowing for absolute quantification of nucleic acids without reliance on a standard curve, effectively circumventing this issue (Quan et al., 2018). Our comparative analysis focuses on assessing the detection capabilities of triplex qPCR and triplex ddPCR. Findings from DNA and spiked milk samples reveal that ddPCR demonstrates heightened sensitivity in comparison to qPCR, capable of detecting nucleic acids at a minimum concentration ten times lower than qPCR. Furthermore, this method holds promise for simultaneous differential diagnosis of mixed infection samples. The extensive genetic similarity and evolutionary connections within MTBC present challenges in identifying unique regions exclusive to *M. bovis* but absent in BCG strains or *M. tuberculosis*. Consequently, our method currently lacks the capability to differentiate between *M. tuberculosis* and *M. tuberculosis*-*M. bovis* co-infection in complex samples.

Through in-depth exploration of the genome sequences of *M. tuberculosis*, *M. bovis*, and BCG strains, this method can enhance future detection capabilities by incorporating additional genetic fragments, such as *mmpS6* (Ma et al., 2022). This particular gene presents in *M. bovis* and BCG strains but absent in *M. tuberculosis*, thereby enabling a more precise differentiation between infections of *M. tuberculosis* or *M. bovis* and *M. tuberculosis* co-infections. However, it should be noted that this approach also falls short in distinguishing between *M. tuberculosis*-*M. bovis* co-infections and *M. tuberculosis*-*M. bovis*-BCG mixed infections. Further analysis and research are warranted to achieve comprehensive differentiation of mixed infections within complex samples.

Compared with traditional diagnostic methods, this established method for identifying *M. tuberculosis*, *M. bovis*, and BCG strains significantly enhances the accuracy and efficiency of diagnosis. It enables the early-stage diagnosis of individual infections, facilitating targeted treatment and vaccination. Furthermore, this study holds the potential for pathogen traceability, epidemic surveillance, and provides a more robust scientific foundation for disease prevention and control.

## Conclusion

In this study, we have successfully developed a triplex ddPCR method for the identification of *M. tuberculosis*, *M. bovis*, and BCG strains, utilizing a three-color ddPCR system. Our established method demonstrates remarkable attributes, including low detection limits (ranging from 3.08 to 4.47 copies per reaction) and excellent specificity. Moreover, it exhibits strong resistance to the presence of milk samples, with lower limits of detection (LODs) reaching the concentrations of 5 × 10^3^ CFU/mL in milk. Notably, this assay allows for the simultaneous detection of three targets in a single sample, introducing a novel and rapid method for sensitive detection, enabling the differentiation of the causative agent in tuberculosis cases.

## Data availability statement

The original contributions presented in this study are included in this article/Supplementary material, further inquiries can be directed to the corresponding authors.

## Author contributions

YQ: Writing – original draft. ML: Writing – review and editing. XS: Writing – original draft. YL: Writing – review and editing. JZL: Writing – review and editing. LH: Writing – review and editing. ZJ: Writing – original draft. FQ: Writing – original draft. WN: Writing – original draft. XY: Writing – original draft. MS: Writing – original draft. WS: Writing – review and editing. JQL: Writing – original draft. SS: Writing – review and editing. HZ: Writing – review and editing. XF: Writing – review and editing.
