# Supplementary material for: Development and evaluation of a triplex droplet digital PCR method for differentiation of M. tuberculosis, M. bovis and BCG
Source: Front Microbiol. 2024 Jun 14;15:1397792. doi: 10.3389/fmicb.2024.1397792 (PMC11211260; doi:10.3389/fmicb.2024.1397792)
Supplement: Supplementary file 1 [file Presentation_1.pdf]

Supplemental Method

1. Nucleic acid extraction method

M. tuberculosis C2 (GCA_000685425.1), *M. bovis* XJ/18/97 (Xu et al., 2021) and BCG Tokyo 172 (AP010918.1) were routinely cultured at 37°C on 7H11 agar or in 7H9 medium for 3 weeks. Genomic DNA was extracted from bacterial culture using an improved nucleic acid extraction method based on the CTAB protocol. In brief, the bacterial culture medium was centrifuged, and then the resulting pellet was frozen overnight and subsequently heated at 80°C to inactivate the bacteria. Following this, SDS solution and proteinase K solution were added to lyse the cell wall. The lysis steps involved the use of a 5M NaCl solution, CTAB solution, and a mixture of chloroform, 3-methylbutan-1-ol, and phenol in a ratio of 25:24:1. Finally, the supernatant was centrifuged, and 3M sodium acetate solution and isopropanol were employed to precipitate the DNA. The DNA was dissolved in nuclease-free water to obtain the final DNA solution.

DNA from the clinical milk, tissue, and swap samples was extracted using a bacterial genome DNA extraction kit (Xi'an Tianlong Science and Technology Co., China) after pretreated, respectively. The milk samples were pretreated by high-speed centrifugation to remove grease and liquid, the tissue samples were pretreated by grinding and centrifugation to collect supernatant, and the swap samples were retreated by centrifugation to collect precipitate after being washed with PBS solution.

Supplemental Tables

Table S1. Bacterial strains and plasmids in this study.

<table>
<tr><th>DNA</th><th>Description</th><th>Source</th></tr>
<tr><td>M. tuberculosis C2</td><td rowspan="13">Preserved DNA solution</td><td rowspan="16">Stored in the National Animal Tuberculosis Reference Laboratory of China Animal Health and Epidemiology Center (Qingdao, China).</td></tr>
<tr><td>M. bovis XJ/18/97</td></tr>
<tr><td>BCG Tokyo 172</td></tr>
<tr><td>Mannheimia haemolytica</td></tr>
<tr><td>Mycoplasma bovis</td></tr>
<tr><td>Haemophilus parasuis.</td></tr>
<tr><td>Escherichia coli</td></tr>
<tr><td>Mycoplasma bovis</td></tr>
<tr><td>Pasteurella multocida</td></tr>
<tr><td>Ochabactum anthropi</td></tr>
<tr><td>Salmonella choleraesuis</td></tr>
<tr><td>Staphylococcus aureus</td></tr>
<tr><td>Brucella melitensis</td></tr>
<tr><td>pRD1</td><td>pUC57 insert CFP-10 to ESAT-6</td></tr>
<tr><td>pRD4</td><td>pUC57 insert Rv0222</td></tr>
<tr><td>pΔRD1</td><td>pUC57 insert Rv3871 and Rv3879c</td></tr>
</table>

Table S2. Oligonucleotide primers and probes used in this study.

Target gene	RD region	Design sequence5'–3'
CFP-10 to ESAT-6	RD1	F1: GGCTTCTGACCCGCTAATAC<br>P1: FAM-ACATTTCCCTGGATTGCGCTTGC-DHQ1<br>R1: TCGTCAAGGAGGGAATGAATG<br>F2: AGTGGAATTTCGCGGGTATC<br>P2: FAM-ACATTTCCCTGGATTGCGCTTGC-DHQ1<br>R2: CTTGGTCAGGGACTGCTTC<br>F3: CCTCGCAAATGGGCTTCT<br>P3: FAM-AGTGGAATTTCGCGGGTATCGAGG-DHQ1<br>R3: GACGTGACATTTCCCTGGATT
Rv0222	RD4	F1: GGAGAAGATCACCGCCAAT<br>P1: VIC-CCACCAAGCGGATTATCACCGAGT-DHQ1<br>R1: TCATCTGCTCAGCGAACATAG<br>F2: GAACAGCGGGATCGGATTT<br>P2: VIC-ATCATCACGATCAACCGCCCGAAA-DHQ1<br>R2: AAGCTGATCCATCGCATCG<br>F3: TAGCGATAGCCATGGAGTTG<br>P3: VIC-TCGATGCTGCGATCGCGTTG-DHQ1<br>R3: CCATTGGCGGTGATCTTCT
Rv3871 and Rv3879c	ΔRD1	F1: GTGGTGGAGCGGATTTGA<br>P1: CY5-

		TTCTGGTCGACGATTGGCACATCA-DHQ2<br>R1: GCAGCTATGCCAGACAGAT<br>F2: GGATTTGACGTCGTGCTTCT<br>R2: CGATCTGGCGGTTTGGG<br>P2: CY5-ATCCAGCATCTGTCTGGCATAGCT-DHQ2<br>F3: TCGCGTTCGTGGTGGAG<br>P3: CY5-ATTTGACGTCGTGCTTCTGGTCGA-DHQ2<br>R3: GACGGGCAGCTATGCCA
