# Supplementary figures and images for: Development and evaluation of a triplex droplet digital PCR method for differentiation of M. tuberculosis, M. bovis and BCG
Source: Front Microbiol. 2024 Jun 14;15:1397792. doi: 10.3389/fmicb.2024.1397792 (PMC11211260; doi:10.3389/fmicb.2024.1397792)

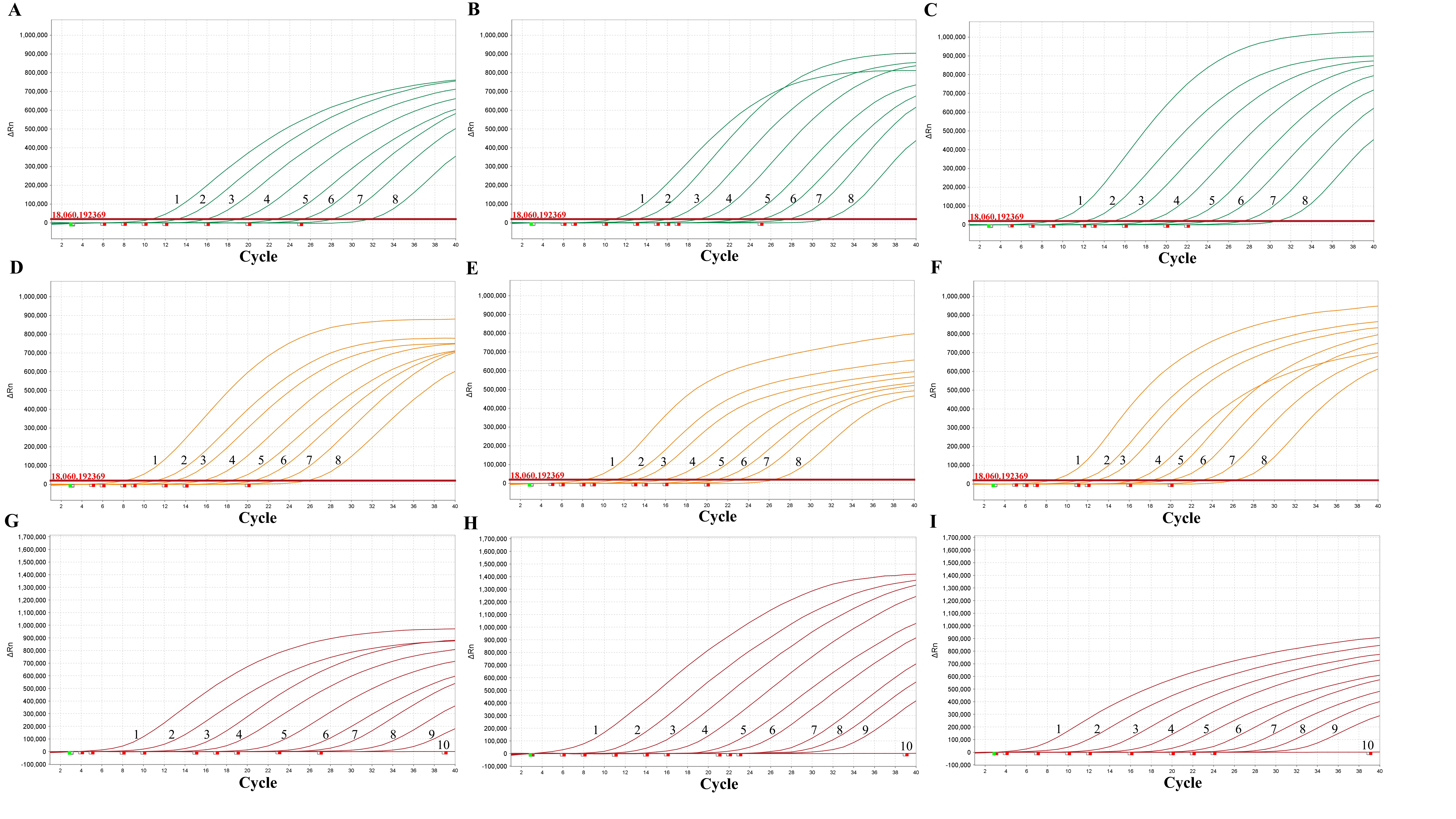

Supplement: Supplementary file 2 [file Image_1.TIF]

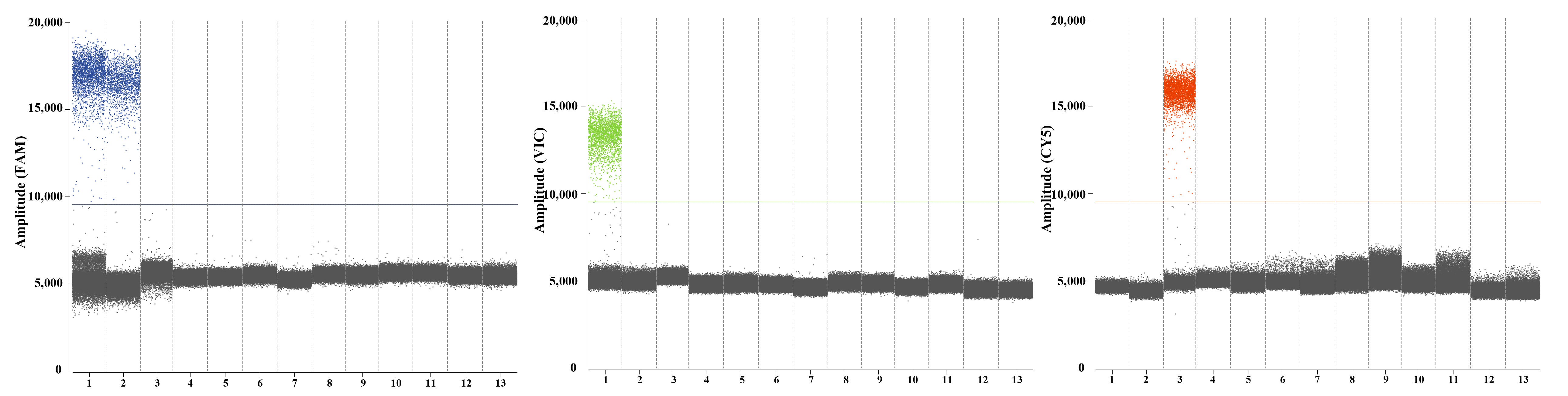

Supplement: Supplementary file 3 [file Image_2.TIF]
